# A Combined Transcriptomic and Proteomic Approach to Reveal the Effect of Mogroside V on OVA-Induced Pulmonary Inflammation in Mice

**DOI:** 10.3389/fimmu.2022.800143

**Published:** 2022-03-18

**Authors:** Tong Dou, Juan Wang, Yisa Liu, Jiangang Jia, Luwei Zhou, Guoxiang Liu, Xiaojuan Li, Mengjie Han, Jiaxun Lin, Fengxiang Huang, Xu Chen

**Affiliations:** ^1^ Department of Pharmacy, Guilin Medical University, Guilin, China; ^2^ Key Laboratory of Pharmacognosy, Education Department of Guangxi Zhuang Autonomous Region, Guilin, China; ^3^ Guangxi Key Laboratory of Molecular Medicine in Liver Injury and Repair, Guilin Medical University, Guilin, China; ^4^ Guangxi Health Commission Key Laboratory of Basic Research in Sphingolipid Metabolism Related Diseases, The Affiliated Hospital of Guilin Medical University, Guilin, China; ^5^ Faculty of Basic Medicine, Guilin Medical University, Guilin, China

**Keywords:** mogroside V, pulmonary inflammation, transcriptomic, proteomic, NF-κB signal pathway, JAK-STAT pathway

## Abstract

Mogroside V is a bioactive ingredient extracted from the natural food *Siraitia grosvenorii* which possesses functions that stimulate lung humidification and cough relief activities, but its underlying mechanisms were rarely studied. To estimate its potential protective effect on ovalbumin (OVA)-induced pulmonary inflammation and understand its system-wide mechanism, integrated omics was applied in this study. Mogroside V effectively reduced the levels of IgE, TNF-α, and IL-5 in OVA-induced mice. The results of RNA-seq and data-independent acquisition proteomics approach revealed that 944 genes and 341 proteins were differentially expressed in the normal control group (NC) and ovalbumin-induced control group (OC) and 449 genes and 259 proteins were differentially expressed between the OC and the group treated with 50 mg/kg mogroside V (MV). After a combined analysis of the transcriptome and the proteome, 93 major pathways were screened, and we discovered that mogroside V exerts an anti-inflammation effect in the lung *via* NF-κB and JAK-STAT, both of which are among the signaling pathways mentioned above. In addition, we found that the key regulatory molecules (Igha, Ighg1, NF-κB, Jak1, and Stat1) in the two pathways were activated in inflammation and inhibited by mogroside V. Thus, mogroside V may be the main bioactivity component in *S. grosvenorii* that exerts lung humidification and cough relief effects.

## Introduction

Asthma is an extensively chronic lung inflammatory disease (1), whose onset is influenced by both endogenous and environmental factors (2). In 2015, about 358 million people worldwide suffered from asthma, which is a significant increase from 183 million in 1990 (3, 4). Allergic asthma is the most general type compared with non-allergic asthma and intrinsic asthma (5). The most important pathological changes in the lungs of asthmatic patients include a large number of lymphocyte and eosinophil infiltrations, excessive mucus secretion of goblet cells, and an increased level of type 2 helper T cell (Th2) cytokine, including interleukin-4 (IL-4), IL-5, and IL-13 in lung tissue (6). These cytokines play an important role in allergic asthma by promoting the production of immunoglobulin E (IgE) as well as a large number of inflammatory mediators and then cause airway inflammation (7, 8) The current treatments for allergic asthma show potential side effects and are not effective for all patients, which caused the incidence and complications of allergic asthma to increase dramatically over the past decade (9), so there is an urgent need for novel treatment strategies for allergic asthma.

In recent years, finding a component with prominent biological activities and minimal side effects from natural herbs used to treat allergic asthma has attracted people’s attention (10, 11). The *Siraitia grosvenorii* fruit (Luo Han Guo in Chinese) is mainly planted in southern Guangxi, especially in Guilin. It has been used as traditional Chinese medicine (TCM) in the treatment of sore throat and lung diseases (12), and its main component, mogroside, is used as a natural sweetener for their high degree of sweetness (13). Mogroside V, a major mogroside in *Siraitia grosvenorii*, is a compound of cucurbitane triterpenoid glycoside which has extensive pharmacological bioactivities, such as anti-tussis, expectorant, anti-inflammatory, and anti-asthmatic (14). It has been developed into many protective products for lung and pharynx diseases. Many studies have shown that mogroside V has the effect of relieving cough, removing phlegm, and humidifying the lung, which is the main reason why Luo Han Guo is made into cough syrups and cough tablets used in clinics (15, 16). However, there are a few studies focusing on the mechanism of mogroside V on pulmonary inflammatory diseases, so it is necessary to comprehensively and systematically understand its effect on lung inflammation disease.

In this study, we aim to demonstrate how mogroside V alleviates ovalbumin (OVA)-induced allergic asthma and to explore the mechanism of reducing the inflammation. Integrated transcriptomic and proteomic analyses are used to identify the potential key factors and specific pathways associated with inflammation that are activated by OVA and modulated by mogroside V.

## Materials and Methods

### Ethics Approval

**Figure 1:**
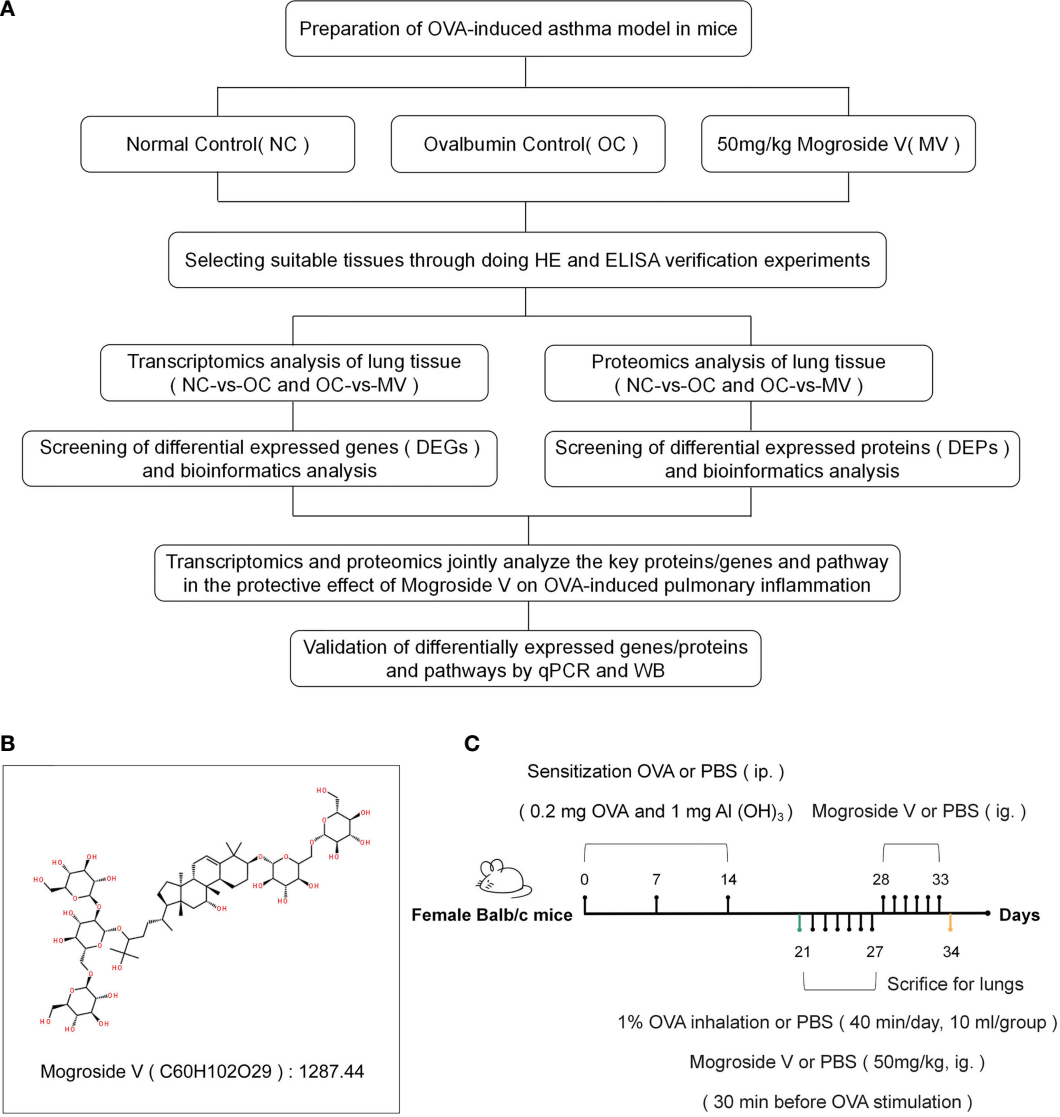
**(A)** Overall approach of the study. **(B)** Molecular structural formula of mogroside V. **(C)** Establishment of a mouse model of asthma and drug administration.

The overall experimental approach, including the animal model, the experimental design, and the combined omics analysis, is shown in **Figure 1A**, and the method of establishing the animal model is shown in **Figure 1C**. The experimental procedures were reviewed and approved by the Experimental Animal Ethics Committee of Guilin Medical College.

### Reagents and Animals

Mogroside V (**Figure 1B**) was purchased from Chengdu Push Bio-Technology Co., Ltd., OVA was from Shanghai Yuanye Bio-Technology Co., Ltd., and 32 6-week-old female BALB/c mice (20 ± 2 g) were bought from Hunan SJA Laboratory Animals Co., Ltd. The animals were fed in a room with controlled temperature (25 ± 2°C) and humidity (50 ± 10°C).

### Model and Drug Administration

We induced allergic asthma in mice with OVA and Al(OH)_3_ (17). Twenty-four mice were distributed into 3 groups—normal control group (NC), ovalbumin-induced control group (OC), and the group treated with 50 mg/kg mogroside V (MV)—with 8 mice in each group (a dose concentration of 50 mg/kg was selected based on our preliminary experiments). The mice in OC and MV were intraperitoneally injected with 0.1 ml of sensitization solution [0.2 mg OVA and 1 mg AL(OH)_3_] on days 0, 7, and 14, respectively, and the mice in NC were intraperitoneally injected with phosphate-buffered saline (PBS) solution. The mice in OC and MV were respectively given every day, at 10 ml/group, 1% OVA spray on days 21–27 with 402AI (Jiangsu Yuyue Medical Equipment & Supply Co., Ltd, China) diaphthography ultrasonography atomizer, and the mice in NC were sprayed with PBS solution (40 min every time). Before OVA was sprayed for 30 min, the drug-treated mice were intragastrically administrated with 50 mg/kg mogroside V, and the NC and OC mice were intragastrically administrated with PBS solution. The MV mice were sequentially given the mogroside V intervention by intragastric administration on days of 28–33, while the NC and OC mice were given PBS in the same way. The mice were sacrificed 24 h after the last oral administration of mogroside V and PBS solution. Lung tissue and blood were collected from each group.

### Histological Analyses

In order to determine whether the model was successfully established, mouse lung tissue was observed by histologic analysis. The lung tissues were fixed in 10% formalin for 48 h, embedded in paraffin, and sliced at 4-μm thickness for histological examination. The sections were stained with hematoxylin and eosin solution (Beijing Solarbio Science & Technology Co., Ltd., China). The recruitment of inflammatory cells in the lung sections was examined under a microscope (OLYMPUS, BX53, Japan), and images were captured.

### Enzyme-Linked Immunosorbent Assay

Besides histological evaluation, the levels of OVA-specific IgE, IL-5, and TNF-α (Dakewe Biotech Co., Ltd., Shenzhen, China) in mice serum were also determined. Blood was collected by cardiac puncture from each group of mice and then left to stand at room temperature for 30 min until it coagulated and stratified. Serum was obtained by centrifugation (Thermo Fisher Scientific, USA) at 3,500 rpm for 15 min and subsequently stored in a refrigerator at -80°C. The expressed levels of IgE, IL-5, and TNF-α in mice were detected by ELISA.

### Transcriptome Analysis

We randomly selected three mice from each group as biological replicates and extracted their total RNA with Trizol reagent kit, whose quality was assessed by Agilent 2100 Bioanalyzer. After that, eukaryotic mRNA and prokaryotic mRNA were enriched. Then, the enriched mRNA was reverse-transcribed into cDNA after they were cut into short fragments. The second-strand cDNA was synthesized and then purified with QiaQuick PCR extraction kit. Lastly, the second-strand cDNA was added with poly(A), ligated to Illumina sequencing adapters, and sequenced using Illumina HiSeq2500 by Gene Denovo Bio-tech Co. (Guangzhou, China). The raw Illumina sequencing data have been uploaded to NCBI (NCBI BioProject: PRJNA777046)

After clean data were obtained, the reads were mapped to the ribosome RNA (rRNA) database and then to the reference genome. The mapped reads of each sample were assembled, each transcription region was calculated, and its expressed abundance and variations were quantified. The calculated gene expression was directly used to analyze the differences in gene expression among these groups. The differentially expressed genes (DEGs) between NC-*vs*.-OC and OC-*vs*.-MV lungs were respectively analyzed. RNA differential expression analysis between two different groups was performed by DESeq2 software. The genes/transcripts with false discovery rate (FDR) ≤0.05 and absolute fold change ≥2 were considered differentially expressed genes/transcripts.

The genes were annotated against GO KEGG and COG/KOG database to obtain their functions. Significant GO functions and pathways were examined within differentially expressed proteins with *q ≤*0.05.

### Proteome Analysis

Three lung tissues were randomly selected as biological replicates from three experimental groups, and the protein was extracted. In brief, lung tissue was transferred into the lysis buffer (2% SDS, 7 M urea, 1 mg/ml protease inhibitor cocktail), and then its supernatant was collected after homogenization and centrifugation at 4°C. We quantitated the total protein in the supernatant using BCA Protein Assay Kit according to the manufacturer’s instructions. The proteins were reduced by dithiotreitol and alkylated by iodoacetamide. Then, the samples were precipitated by acetone at -20°C overnight, washed twice with cold acetone, and resuspended in ammonium bicarbonate (50 mM). After that, the proteins were digested with trypsin (Promega, Madison, WI, USA) at 37°C for 16 h.

The peptide mixture was fractionated by high-pH reversed-phase separation, then re-dissolved in solvent A (0.1% formic acid in water), and analyzed by online nanospray LC–MS/MS on an Orbitrap Fusion Lumos coupled to EASY-nLC 1200 system (Thermo Fisher Scientific, MA, USA), thereby generating raw data for mass spectrometry detection (ProteomeXchange ID: PXD029664). Furthermore, 3 μl of the peptide sample was loaded onto the analytical column (Acclaim PepMap C18, 75 μm × 25 cm) with a 120-min gradient, from 5 to 35% B (B: 0.1% formic acid in acetonitrile). The column flow rate was maintained at 200 nl/min, with a column temperature of 40°C. The electrospray voltage of 2 kV *versus* the inlet of the mass spectrometer was used.

The mass spectrometer was run under data-independent acquisition (DIA) mode and automatically switched between MS and MS/MS modes. The raw data of DIA were processed and analyzed by Spectronaut X (Biognosys AG, Switzerland) with default parameters. All selected precursors passing the filters were used for quantification. The average top 3 filtered peptides which passed the 1% *Q* value cutoff were used to calculate the major group quantities. After subjecting to Student’s *t*-test, differentially expressed proteins were filtered if their *p <*0.05 and absolute fold change ≥1.5.

The proteins were annotated against GO KEGG and COG/KOG database to obtain their functions. Significant GO functions and pathways were examined for differentially expressed proteins with *p ≤*0.05.

### mRNA and Protein Correlation Analyses

Firstly, we identify differentially expressed genes and proteins among these groups. mRNA with fold change ≥2 and FDR <0.05 was identified as significant DEG, while protein with fold change >1.5 and *p <*0.05 was identified as significant differentially expressed proteins (DEP).

Then, the association between genes and proteins was quantitatively analyzed. The genes/proteins and differential genes/differential proteins detected in the transcriptome and the proteome were counted separately, and a Venn diagram was plotted using this data. A nine-quadrant map analysis was drawn to show the correlation between genes and proteins. The analysis was performed by R language (version 3.5.1).

To assess functional enrichment, Gene Ontology (GO) biological processes terms and Kyoto Encyclopedia of Genes and Genomes (KEGG) pathway analysis of mRNAs were employed in the nine-quadrant map. A correlation analysis between GO function (http://www.geneontology.org/) and KEGG pathway information (http://www.kegg.jp/kegg/) in the transcriptome and the proteome was performed, and the similarities and differences between gene function and metabolic pathway in the two groups were compared.

### Quantitative Reverse Transcription PCR

We used Trizol (TIANGEN, China) to obtain total RNA from the lung. Nanodrop One was used to analyze the total mRNA concentration (Thermo Fisher Scientific, USA). cDNA was synthesized from 2 μg of total RNA using a ReverTra Ace-cDNA Synthesis kit (Promega, USA). Gene expression analysis was performed on Applied Biosystems™ 7500 Fast Real-Time PCR system (Thermo Fisher Scientific, USA). The primer sequences used for the gene expression analysis are shown in **Table 1**. The 2^-ΔΔCt^ method was used to analyze the mRNA fold change by ABI 7500 fast v2.0.1.

**Table 1:** Sequences of forward and reverse primers used in qRT-PCR.

RNA species	Primer pairs
JAK	Forward:5′-TGAAAAGTCTGAGGTATTGGG-3′ Reverse:5′-ATCTGCTTCTTGAGGTGGTT-3′
STAT1	Forward:5′-GTCTCAGTGGTACGAACTTCAG-3′ Reverse:5′-GTGCCAGGTACTGTCTGATTT-3′
Igha	Forward:5′-CTCCTCCTTGTCCTCCTTGT-3′ Reverse:5′-AGACAGCTCCCTCAGGATTT-3′
Ighg1	Forward:5′-TGGAAACAGGGTGACCAGTA-3′ Reverse:5′-CGCTGACATTGGTGGGTTTA-3′
NF-κB	Forward:5′-GTGGGGACTACGACCTGAATG-3′ Reverse:5′-GGGGCACGATTGTCAAAGATG-3′
IkB	Forward:5′-ACCTGGTGTCACTCCTGTTGA-3′ Reverse:5′-CTGCTGCTGTATCCGGGTG-3′
β-Actin	Forward:5′-AAAGACCTGTACGCCAACAC-3′ Reverse:5′-GTCATACTCCTGCTTGCTGAT-3′

### Western Blotting

Lung tissue protein was extracted by RIPA buffer (Beijing Solarbio Science & Technology Co., Ltd., China). After the quantification of proteins, they were separated by sodium dodecyl sulfate–polyacrylamide gel electrophoresis and transferred on NC membranes. The membranes were blocked with 5% skim milk and then incubated with primary antibodies against the target proteins (Abcam, Cambridge, UK; Abmart Inc., Shanghai, China) at 4°C overnight. Then, the membranes were incubated with secondary antibodies (Beijing Emarbio Science & Technology Co., Ltd.; China; anti-rabbit, Thermo Fisher Scientific, Inc., USA) for 1 h. The membranes were treated with enhanced chemiluminescence detection reagent (Biosharp, Shanghai, China), and the protein bands were visualized using Biorad Chemidoc XRS+ ChemiLuminescence Imaging System (Bio-Rad, USA). The relative band density was determined by Image J (National Institutes of Health, Bethesda, MD, USA).

### Statistical Analysis

Data were determined by one-way analysis of variance (ANOVA) with SPSS 22.0 (SPSS, Chicago, IL, USA). The results are expressed as means ± SEM, and the comparison between different groups was analyzed by using *t*-test. For all tests, *p <*0.05 was seen as statistically significant.

## Results

### Effect of Mogroside V on the Pathological Changes of Lung Tissue in Asthmatic Mice

**Figure 2:**
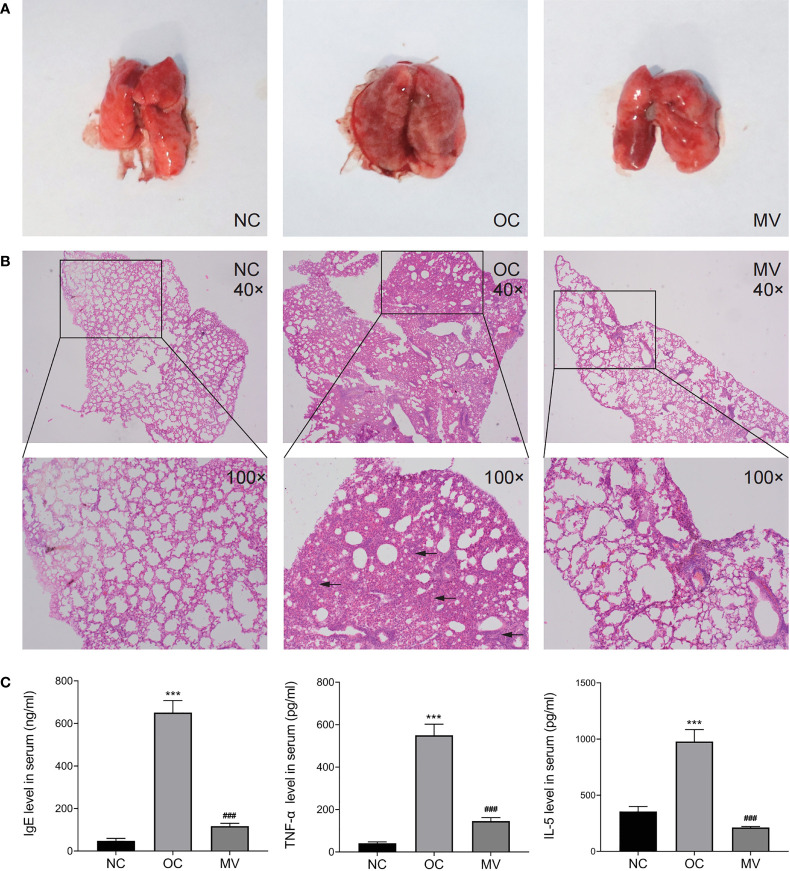
**(A)** Representative histomorphological observation of lungs from different groups. **(B)** Hematoxylin and eosin-stained mouse lung sections from the normal control group (NC), the ovalbumin control group (OC), and the group treated with 50 mg/kg mogroside V (MV). The original magnification was ×40 (upper panels). Inserts show magnified regions at ×100 (lower panels). **(C)** Mogroside V treatment attenuates the production of Th1/Th2 cytokines as indicated by the levels of IgE, TNF-α, and IL-5 determined by ELISA kits. All values are mean ± SEM (*n* = 3) by one-way ANOVA. ***P < 0.001 NC *vs*. OC group; ###*P* < 0.001 OC *vs*. MV group.

The histopathological observation (**Figure 2A**) of the lung showed that, compared with NC, OC showed obvious hyperemia and increased volume in the lungs. However, these conditions were significantly improved in the MV comparing with the OC. The results of lung histopathology (**Figure 2B**) showed that OVA induced alveolar wall thickening and massive infiltration of inflammatory cells. Compared with OC, the extent and the intensity of infiltration in MV were less than those of it, suggesting that the construction of a model was successful and that mogroside V could alleviate the pulmonary inflammatory response induced by OVA in mice.

### Mogroside V Modifies Th2 Cytokines in OVA-Exposed Mice Serum

Given that elevated Th2 cytokines are associated with the pathogenesis of allergic reactions, inflammatory cytokines were examined. As shown in **Figure 2C**, the IgE, TNF-α, and IL-5 levels were increased to be higher in OC than in NC, which was significantly reversed by mogroside V (*p* < 0.05).

### Transcriptomics Analysis

**Figure 3:**
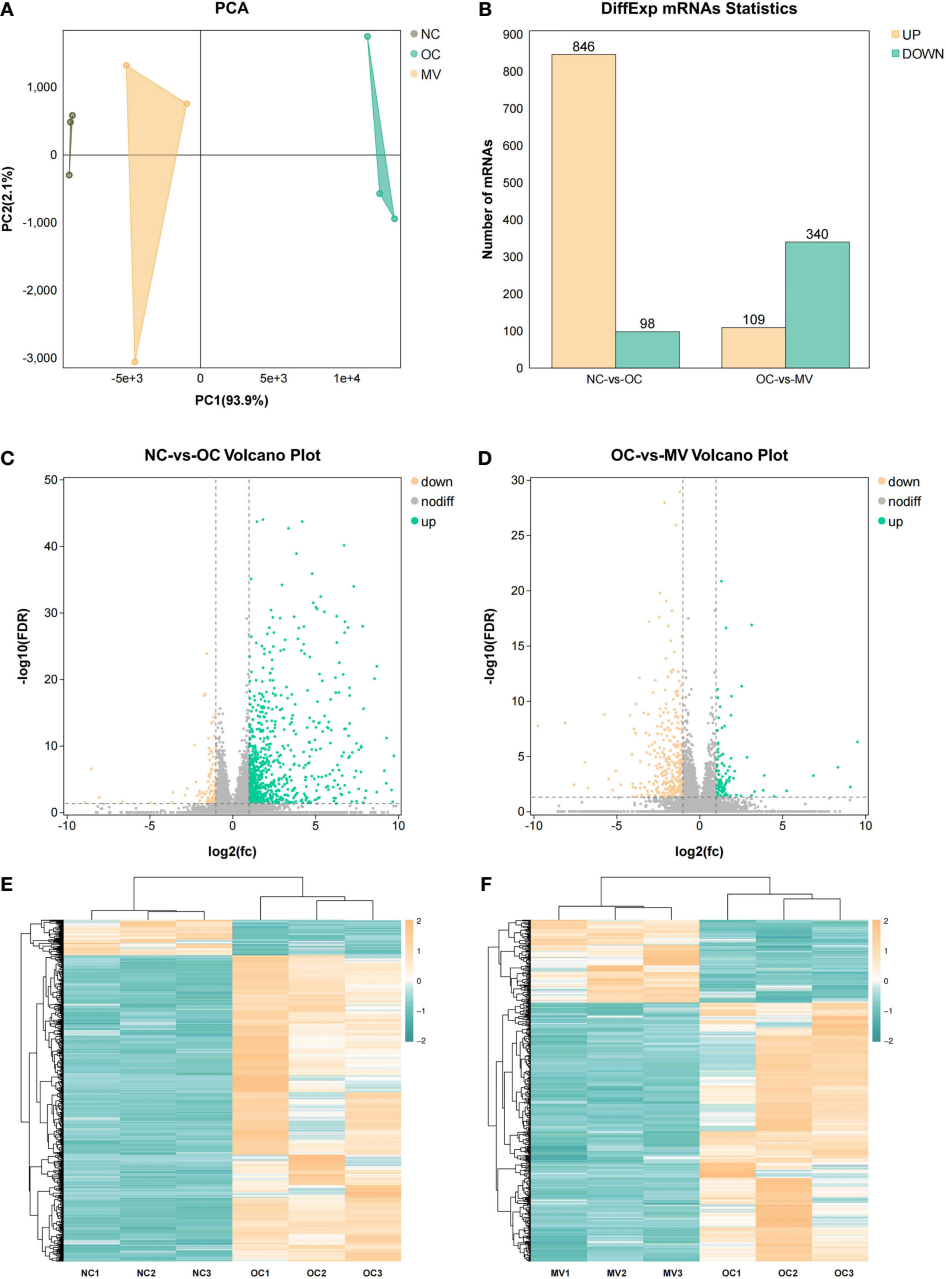
Differential analysis in mRNA expression levels between the normal control group or the group treated with 50 mg/kg mogroside V and the lungs of the ovalbumin control group. **(A)** Principal component analysis of RNA-seq data. The points represent biological replicates. **(B)** Histogram of differential gene expression among different groups. **(C, D)** Volcano plots showing the relative abundances of transcripts. The transcripts were considered differentially expressed at fold change >2 and with statistical significance (*p* < 0.05) between groups. The gray points represent the genes in which no statistically significant differences were observed. The red points represent the downregulated genes, and the green points represent the regulated genes that were statistically significant. **(E, F)** Heat map of gene expression among the different groups.

To determine the gene expression changes, an RNA-seq analysis was performed to investigate the transcriptome in asthmatic mice. The principal component analysis (PCA) results showed large variations in the transcripts between NC and OC (**Figure 3A**), and there was less variation between MV and NC. The PCA indicated a distinct directionality between the two groups based on similarities in gene expression. These results indicated that MV treatment reduced the transcript variations caused by OVA. Following this analysis, the DEGs that were caused by different treatments were described by volcano plots (**Figures 3C, D**). When the RNA-seq results were compared between the NC-*vs*.-OC and OC-*vs*.-MV groups, respectively, 22,298 genes were detected by RNA-seq. When the RNA-seq results were compared between the NC and OC group, 944 genes were found to be differentially expressed, in which 846 genes were upregulated and 98 genes were downregulated (**Figure 3B**). Compared with OC, a total of 449 differentially expressed genes were screened out in MV, including 109 upregulated genes and 340 downregulated genes (**Figure 3B**). The gene expression pattern between NC-*vs*.-OC and OC-*vs*.-MV is shown as heat map in **Figures 3E, F**.

### Alterations of Molecules on the Proteomic Level

**Figure 4:**
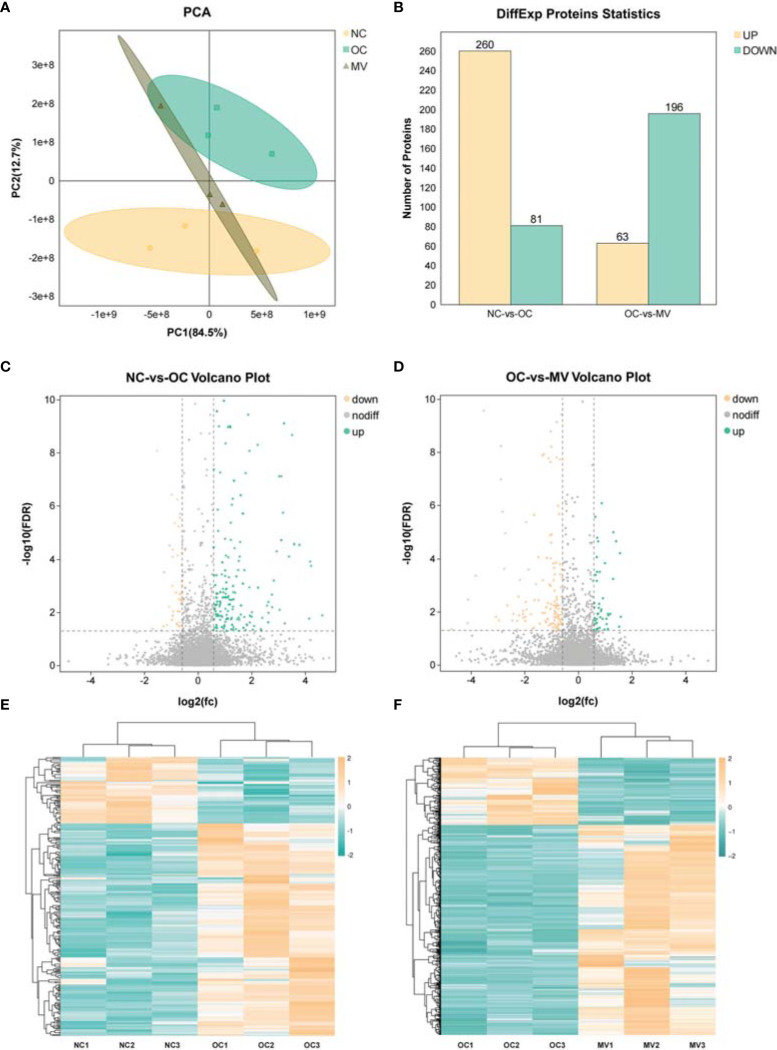
Differential analysis in protein expression levels between the normal control group (NC) or the group treated with 50 mg/kg mogroside V (MV) and the lungs of the ovalbumin control group (OC). **(A)** Principal component analysis of proteomic data in NC, OC, and MV, including all three groups. These dots represent biological replication. **(B)** Histogram of differential protein expression between different groups. **(C, D)**. The volcano map shows the relative abundance of proteins. No statistically significant differences were observed in the proteins represented by the gray dots. The red dots represent downregulated proteins, and the green dots represent upregulated proteins, which are statistically significant. **(E, F)** Heat map of protein expression among the different groups.

Proteomic technology can be used to learn the large-scale structure and function of proteins in complex biological samples. In this study, we initially assessed the clustering of the groups *via* PCA (**Figure 4A**). The PCA results showed that the samples were divided into 2 main clusters, which indicated an obvious differential expression of protein after having been induced by OVA, and MV treatment decreased OVA’s effect on mouse lung. The DEPs induced by different treatments were described by a volcano map (**Figures 4C, D**). In total, 341 and 259 proteins had differential expression between NC-*vs*.-OC and OC-*vs*.-MV lungs, respectively. Among the 341 differential proteins screened out in OC, 260 proteins were upregulated and 81 proteins were downregulated (**Figure 4B**), while 63 proteins were upregulated and 196 proteins were downregulated in OVA-exposed mice treated with MV (**Figure 4B**). The protein expression pattern between NC-*vs*.-OC and OC-*vs*.-MV is shown as heat map in **Figures 4E, F**.

### Common Trends of DEPs and DEGs

**Figure 5:**
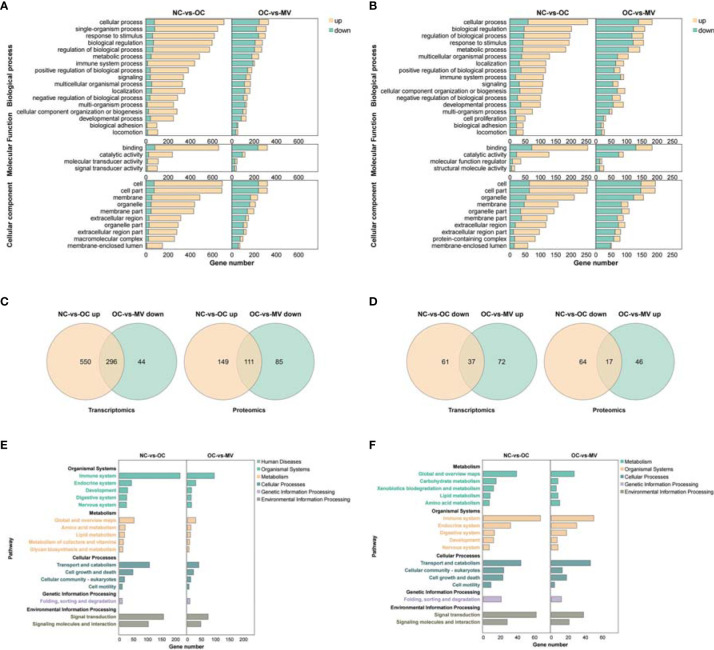
**(A)** Overlapped GO in the normal control group (NC) vs. the ovalbumin control group (OC) (right) and OC vs. the group treated with 50 mg/kg mogroside V (MV) (left) at the transcriptomic level. **(B)** Overlapped GO in the NC-vs.-OC group (right) and OC-vs.-MV group (left) at the proteomic level. **(C)** Overlapped mRNAs (left) and proteins (right) between the upregulated NC vs. the OC group and decreased OC-vs.-MV group. **(D)** Overlapped mRNAs (left) and proteins (right) between the decreased NC-vs.-OC group and upregulated OC-vs.-MV group. **(E)** Overlapped KEGG pathways in the NC-vs.-OC group (right) and OC-vs.-MV group (left) at the transcriptomic level. **(F)** Overlapped KEGG pathways in the NC-vs.-OC group (right) and OC-vs.-MV group (left) at the proteomic level.

Interestingly, we found that there are more upregulated genes than downregulated genes in the comparisons of OC and NC at each pathway, and more genes were decreased in the MV group than in the OC group (**Figure 5A**). These changes were consistent with the trend at the proteomic level (**Figure 5B**). These results reminded us that there may be some common genes which were changed obviously by OVA and then restored by mogroside V. We found that there were 296 DEGs and 111 DEPs that were downregulated in the MV mice compared to the OVA-induced mice but upregulated in the OVA-induced mice compared to the control mice (**Figure 5C**). There were 37 DEGs and 17 DEPs that were upregulated in the MV group compared to the OVA group but downregulated in the OVA group compared to the control group (**Figure 5D**).

### KEGG Pathway Enrichment Analysis for Common Change of DEGs and DEPs

It is interesting that the immune system, as part of the organismal system, is a very important pathway in inflammation. In addition, a large number of DEPs/DEGs are enriched into these pathways, which are associated with the immune system, transport and catabolism, and signal transduction (**Figures 5E, F**).

Furthermore, we found that there are 34 common DEGs/DEPs in the NC-*vs*.-OC and OC-*vs*.-MV comparisons (**Supplementary Table S1**) with R program. In addition, we also found that immunoglobulin heavy constant gamma 1 (Ighg1), immunoglobulin heavy constant alpha (Igha), and signal transducers and activators of transcriptions 1 (Stat1) belong to the NF-KB and JAK-STAT1 pathways, which play important roles in the immune system. Moreover, these three DEGs/DEPs had a significantly different expression in the mogroside V-treated group compared with the OC group.

In order to screen out the key pathways by which mogroside V improved the OVA-induced asthma, we analyzed the KEGG results of NC-*vs*.-OC and OC-*vs*.-MV pairs with R program and obtained 146 target common pathways (**Supplementary Table S2**). We focus on the NF-κB and JAK-STAT pathways. The NC-*vs*.-OC group and the OC-*vs*.-MV group jointly enriched the associated genes, as shown in **Table 2**. The combined analysis of NC-*vs*.-OC and OC-*vs*.-MV resulted in common genes/proteins, namely, Igha, Ighg1, and Stat1.

**Table 2:** Conjoint analysis results of NC-*vs*.-OC & OC-*vs*.-MV pathway (only included in this paper).

Pathway	Diff-gene	Diff-pro	Common	Symbol	Cor. *P*-value
NF-κB	62 (22)	10 (6)	8 (4)	Igha, Ighg1, Igha, Ighg1	0.01697 (1.11E-16)
JAK-STAT1	14 (9)	8 (3)	3 (3)	Stat1, Stat1, Stat1	0 (0)

Group OC-vs.-MV is shown in brackets.

### The Correlation Between Transcriptome and Proteome

**Figure 6:**
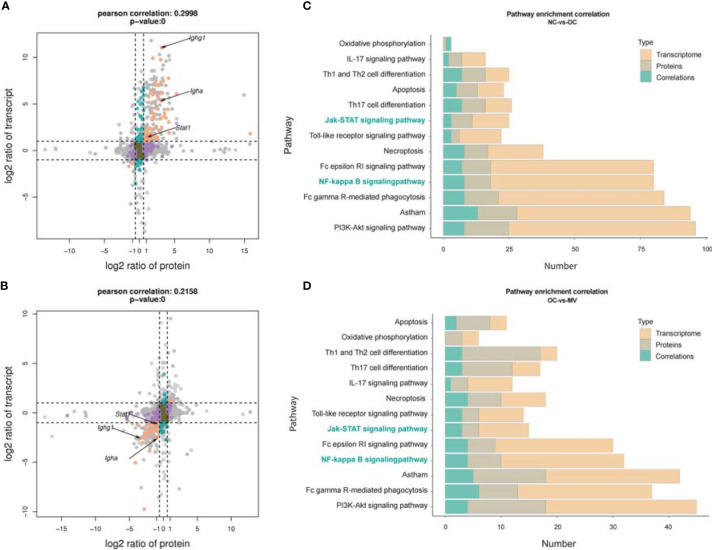
Correlation between transcriptome and proteome. **(A)** Normal control group (NC) *vs*. ovalbumin control group (OC). **(B)** OC *vs*. the group treated with 50 mg/kg mogroside V (MV), nine-quadrant graph (considering the statistical significance, *p*) [The abscissa is the fold change of the protein (take log2), the ordinate is the fold change of the transcriptome (take log2), and at the top of the figure is the Pearson correlation and statistical significance, *p*, associated with the transcriptome and the proteome. Each dot represents a gene/protein: the brown dots represent non-different proteins and genes, and the red dots represent the same or opposite trend of changes in genes and proteins, and the blue dots indicate the differential expression of genes but non-differential expression of proteins]. Histogram of the number of genes on the pathway overlapped by NC *vs*. OC **(C)** and OC *vs*. MV **(D)** [The number of differentially expressed mRNA (red), protein (gray), and associated genes (blue), respectively, is annotated on the pathway].

Integrated analysis was performed to explore the concordance between mRNA and protein levels. Both upregulated mRNA and protein levels (log2 fold change >1.0) were selected in NC-*vs*.-OC groups. Furthermore, 318 mRNA and protein levels were significantly changed, and their changes were correlated with the mRNA and protein levels (**Figure 6A**), so 203 mRNA and protein were downregulated (**Figure 6B**). To screen out the pathways that may play important roles with mogroside V, a pathway enrichment analysis, based on the KEGG database, was performed for these two quadrants, and we got 93 pathways. They were found to be significantly relevant to immunity and inflammation, from which 13 pathways with strong associations are shown in **Figures 6C, D**, which include the IL-17 signaling pathway, Th1 and Th2 cell differentiation, Th17 cell differentiation, Fc gamma R-mediated phagocytosis, Fc epsilon R1 signaling pathway, and PI3K-Akt signaling pathway, *etc.* It is interesting to note that the NF-κB signaling pathway and the JAK-STAT signaling pathway are among the classic inflammatory pathways. According to the data analysis, Igha, Ighg, and Stat1 were significantly enriched in the NF-κB signaling pathway and the JAK-STAT signaling pathway (**Table 3**).

**Table 3:** Expression of differentially expressed genes and differentially expressed proteins with the same trend for proteome and transcriptome in the comparison of NC-*vs*.-OC and OC-*vs*.-MV.

Gene/protein name	mRNA		Protein	
	NC-*vs*.-OC (Q3)	OC-*vs*.-MV (Q7)	NC-*vs*.-OC (Q3)	OC-*vs*.-MV (Q7)
	Fold change	Fold change	Fold change	Fold change
Igha	40.9305263157894	0.161858968674289	8.61753710446117	0.15143586792309
Ighg1	2133.17689906348	0.174203883641961	9.25604629725371	0.0868820096536416
Stat1	2.76712518144743	0.462405075939248	2.28668135597791	0.500100829936149

### Mogroside V Modulates the Validation of JAK-STAT1/NF-kB Signaling Pathways

**Figure 7:**
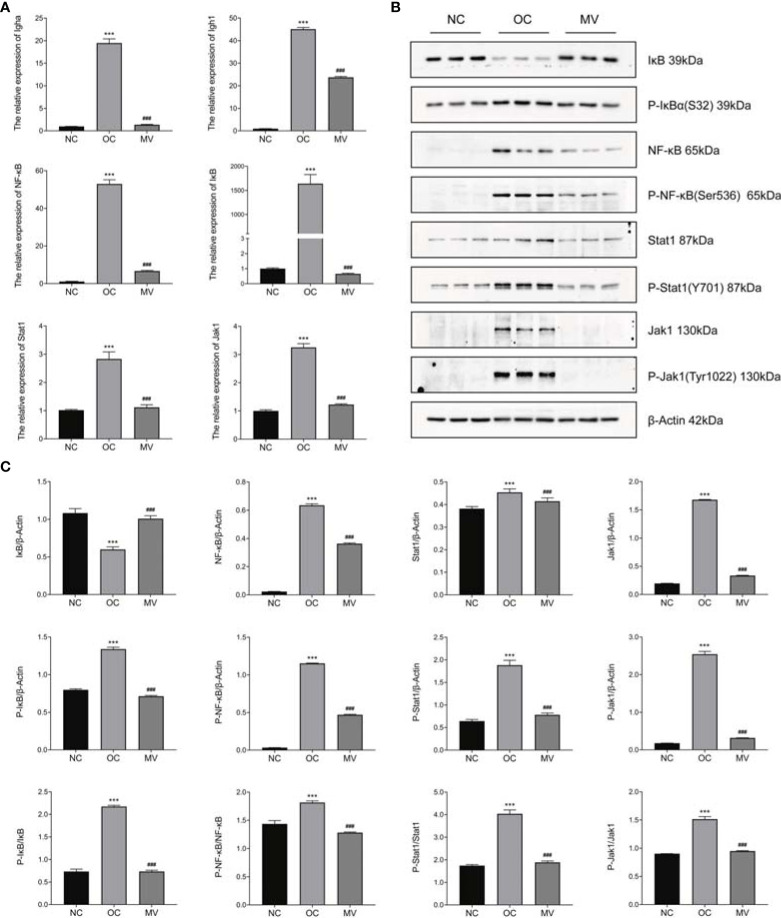
Expression levels of Igha, Ighg1, NF-κB, IκB, Jak1, and Stat1 by qRT-PCR and Western blot (WB). **(A)** The relative mRNA expression levels of Igha, Ighg1, NF-κB, IκB, Jak1, and Stat1 were measured by qRT-PCR and normalized to that of β-actin. **(B)** Western blot. **(C)** The relative protein expression levels of Igha, Ighg1, NF-κB, IκB, Jak1, and Stat1 were measured by WB and normalized to that of β-actin. All values are mean ± SEM (*n* = 3) by one-way ANOVA. ***P < 0.001, normal control group *vs*. ovalbumin control group (OC); ^###^P < 0.001, OC vs. the group treated with 50 mg/kg mogroside V (MV).

From the correlation between the transcriptome and the proteome, we found that the JAK-STAT1/NF-kB signaling pathway was correlated with a protective effect of mogroside V on OVA-induced mouse lung, and this deserves further research. Then, we verified the factors Igha, Ighg1, NF-κB, IκB, Jak1, and Stat1 in the two pathways by RT-PCR and Western blot (WB). As shown by the RT-PCR result, OVA significantly increased the expression of Igha, Ighg1, NF-κB, IκB, Jak1, and Stat1, which was reversed by mogroside V (**Figure 7A**). The results were in accordance with our transcriptome analysis. Moreover, as exhibited in **Figures 7B, C**, compared with NC, the levels of phosphorylated NF-κB (P-NF-κB), phosphorylated IκB (P-IκB), phosphorylated Jak1 (P-Jak1), and phosphorylated Stat1 (P-Stat1) in OC were significantly increased, which means that the inflammation-related pathway JAK-STAT1/NF-kB was activated by OVA, while the activation effect of OVA on the JAK-STAT1/NF-kB pathway was inhibited by mogroside V in mouse lung. These data indicated that mogroside V alleviates inflammation in asthmatic mice by inhibiting the activation of the NF-kB pathway and the JAK-STAT1 pathway.

## Discussion

*S. grosvenorii* is a famous Chinese traditional medicine used in the treatment of cough, sore throat, asthma, and constipation. Seldom are studies that focus on the mechanism of *S. grosvenorii* in the context of its traditional use to humidify the lung and relieve cough. In this study, a systems-level analysis was applied to explore the mechanism of mogroside V in improving OVA-induced pulmonary inflammation in mice.

Asthma and associated type 2 inflammation are driven by both the innate (type 2 innate lymphoid cells) and adaptive [T helper type 2 (Th2) cells] immune systems, characterized by the release of cytokines such as interleukin (IL)-4, IL-5, and IL-13, key pathophysiologic characteristics of asthma (18, 19). Allergic asthma is associated with increased levels of IgE. The activation of the IgE receptor induces TNF-α release in lung tissue and, in turn, upregulates the eosinophil TNF levels (20). Moreover, in animal asthma models, IgE production was also enhanced by IL-5, which is primarily produced by Th2 cells (21), thereby promoting eosinophil maturation, activation, and trafficking (22) and then promoting the degranulation of mast cells and basophils and the development of allergic reactions in asthma. These factors work together to promote the progress of inflammation in allergic asthma mice. In the present study, a widely used allergic asthma mouse model has been established, in which the levels of IgE, TNF-α, and IL-5 are successfully upregulated by OVA compared with the NC control. Then, we found that mogroside V alleviated the lung’s inflammatory response by diminishing the recruitment of inflammatory cells and decreasing the increased levels of IgE, TNF-α, and IL-5. These findings revealed that mogroside V exerted an anti-inflammatory activity on OVA-induced lung inflammation.

High-throughput molecular biological techniques, such as transcriptomic and proteomic approaches, have been used to explore complex biological processes and the function of natural drugs in systematic biology. More and more studies revealed that combining transcriptomic and proteomic techniques to analyze the complex molecular mechanism of natural herbal extracts is a promising approach (23, 24). Initially, the direct differentially expressed genes and proteins were screened out after OVA stimulation and mogroside V intervention. We found that 316 genes and proteins that were significantly upregulated in the model group were obviously ameliorated by mogroside V treatment and that 201 genes and proteins that were significantly downregulated in the model group were obviously ameliorated by mogroside V treatment. After enriching these overlapping differential genes/proteins to KEGG, we found that there were 93 pathways changed by mogroside V after OVA intervention. These pathways are mainly related to asthma, inflammation, and immunity. Interestingly, the anti-inflammatory activity of mogroside V was involved in multi-pathways, including the common inflammation pathways of NF-κB and JAK-STAT1. In previous studies, mogroside V has never been reported to reduce pulmonary inflammation through NF-κB and JAK-STAT.

Stat1 acts as a new molecular target for the reported anti-inflammatory therapy, playing an important role in the pulmonary inflammation process as has been identified in various organs (25). As an important transcription factor in the JAK/STAT signaling pathway, Stat1 plays a key role in inflammation reaction (26). Stat1 can be activated by its upstream phosphorylated JAK1 and JAK2 and then transported into the nucleus to promote the expression of proinflammatory factors, including TNF-α (27, 28). As the commonly identified factor regulated in mogroside V-treated mice by transcriptomic and proteomic analyses, it is a key factor in triggering lung inflammation (29). Our result by ELISA analysis showed that mogroside V significantly reduced the production of TNF-α and reduced the phosphorylated level of JAK1 and Stat1 *in vitro*. A previous study demonstrated that mogroside V ameliorates OVA-induced pulmonary inflammation *via* inhibiting the JAK-STAT1 pathway in mice.

Similar to Stat1, NF-κB is also an important transcriptional regulator that modulates the immune and inflammatory responses (8). Ighg1 and Igha, two factors that are associated with the NF-κB signaling pathway, were also significantly augmented in the OVA-induced group, and they were obviously mitigated by the treatment with mogroside V. NF-κB pathway is a promising inflammatory pathway for drug intervention in the treatment of a variety of lung diseases, including asthma (30–32). NF-κB is a nuclear transcription factor which is closely related to asthma. Studies have shown that activated NF-κB promotes various inflammatory gene transcriptions that are related to asthma. Pgaek et al. and Barnes et al. have also shown that NF-κB activity is enhanced in experimental models and patients of asthmatic allergic inflammation (33, 34). Under non-inflammatory conditions, the inactivation of NF-κB was sequestered in the cytoplasm by the NF-κB (IκB) inhibitor. When the cells are stimulated by external stimuli such as OVA, TNF-α, or IL-1, the activation of NF-κB is started by IκB kinase, which induces the rapid release of NF-κB by degrading the cytoplasmic IκB protein (35, 36). We found that the protein levels of IκB were decreased in the OVA-induced group and those of phosphorylated NF-κB (P-NF-κB) and phosphorylated IκB (P-IκB) were increased in the OVA-induced group similarly to those at the gene level, while the levels of P-NF-κB and P-IκB were decreased by mogroside V. Our omics and RT-PCR and WB results validated that mogroside V inhibited the activation of the NF-κB and JAK-STAT1 pathways, thereby exerting an anti-inflammatory effect in OVA-induced mice.

Stat1 is very important for NF-kB nuclear translocation (37), and its elysine acetylation may be necessary for the release of NF-kB from IκB inhibition in response to external stimuli (38). In this study, we demonstrated the protective effect of the mechanism of mogroside V on OVA-induced pulmonary inflammation. Evidence exhibiting the cross-talk association of Stat1 with the NF-kB pathway in OVA-induced asthma and how mogroside V treated the cells should be further investigated.

Based on integrative analysis, we have identified many pathways associated with immunity and inflammation, such as IL-17 signaling pathway, Th1 and Th2 cell differentiation, Th17 cell differentiation, Fc gamma R-mediated phagocytosis, Fc epsilon R1 signaling pathway, PI3K-Akt signaling pathway, *etc.*, from which it would be good to explain the new mechanism of action of mogroside V as mediated by Th1, Th2, and Th17, so the anti-inflammation effect of mogroside V may be closely related to immunomodulation, which may be regulated by innate immune cell activity *via* TLR signaling. However, an analysis of the functional changes based on immunological cells is necessary, so the effect of mogroside V on immune cells needs further study.

## Conclusion

In conclusion, this study elucidated the role that mogroside V plays in humidifying the lung and relieving cough—which provided a scientific explanation for the clinical use of *S. grosvenorii* contained in TCM products—and promoted the discovery of herb-derived anti-inflammation natural products. Our study has important significance in terms of providing a more comprehensive understanding of the mechanism of *S. grosvenorii* in alleviating OVA-induced pulmonary inflammation.

## Data Availability Statement

The datasets presented in this study can be found in online repositories. The names of the repository/repositories and accession number(s) can be found in the article/**Supplementary Material**.

## Ethics Statement

The animal study was reviewed and approved by the Experimental Animal Ethics Committee of Guilin Medical College.

## Author Contributions

JW and XC provided experimental ideas. TD, YL, and LZ raised the animals. TD, JW, JJ, and GL completed the omics analysis. TD, XL, MH, JL, and FH participated in completing the experiments. TD edited the graphics. TD and JW wrote and revised the manuscript. TD, JW, and XC confirmed the authenticity of all the raw data. All authors contributed to the article and approved the submitted version.

## Funding

This research was funded by the Project of Guangxi Special Fund Project for Innovation-Driven Development (GuikeAA19254025), the National Natural Science Foundation of China (grant nos. 81760443, 81760663, 82160768, and 82002822), the Natural Science Foundation of Guangxi Province (grant number GuikeZD20302006), the Fourth Batch of Bagui Scholars’ Special Funds for 2017 [grant no. (2017) 143], the Science and Technology Planned Project in Guilin (20190206-1), the Guangxi Distinguished Experts Special Fund (2019B12), the Medical High Level Talents Training Plan in Guangxi (G202002005), and the Construction Fund of Guangxi Health Commission Key Laboratory of Basic Research in Sphingolipid Metabolism Related Diseases (ZJC2020005). We thank all about of them for institutional and financial support.

## Conflict of Interest

The authors declare that the research was conducted in the absence of any commercial or financial relationships that could be construed as a potential conflict of interest.

## Publisher’s Note

All claims expressed in this article are solely those of the authors and do not necessarily represent those of their affiliated organizations, or those of the publisher, the editors and the reviewers. Any product that may be evaluated in this article, or claim that may be made by its manufacturer, is not guaranteed or endorsed by the publisher.
