# Supplementary figures and images for: A Combined Transcriptomic and Proteomic Approach to Reveal the Effect of Mogroside V on OVA-Induced Pulmonary Inflammation in Mice
Source: Front Immunol. 2022 Mar 18;13:800143. doi: 10.3389/fimmu.2022.800143 (PMC8972588; doi:10.3389/fimmu.2022.800143)

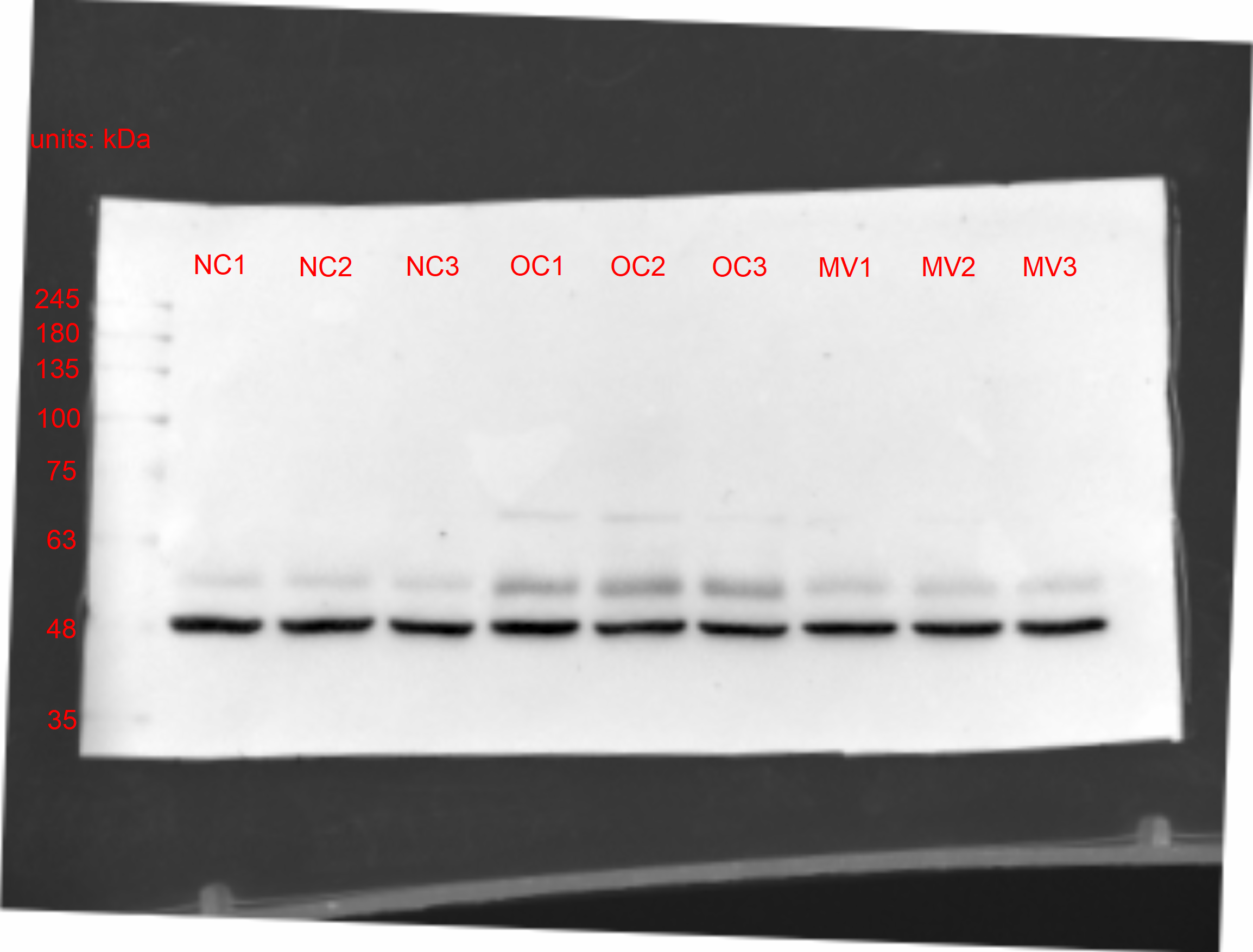

Supplement: Supplementary file 1 [file DataSheet_1.zip › WB╘¡╩╝╩2╛▌-TIFF/A-HB-.tif]

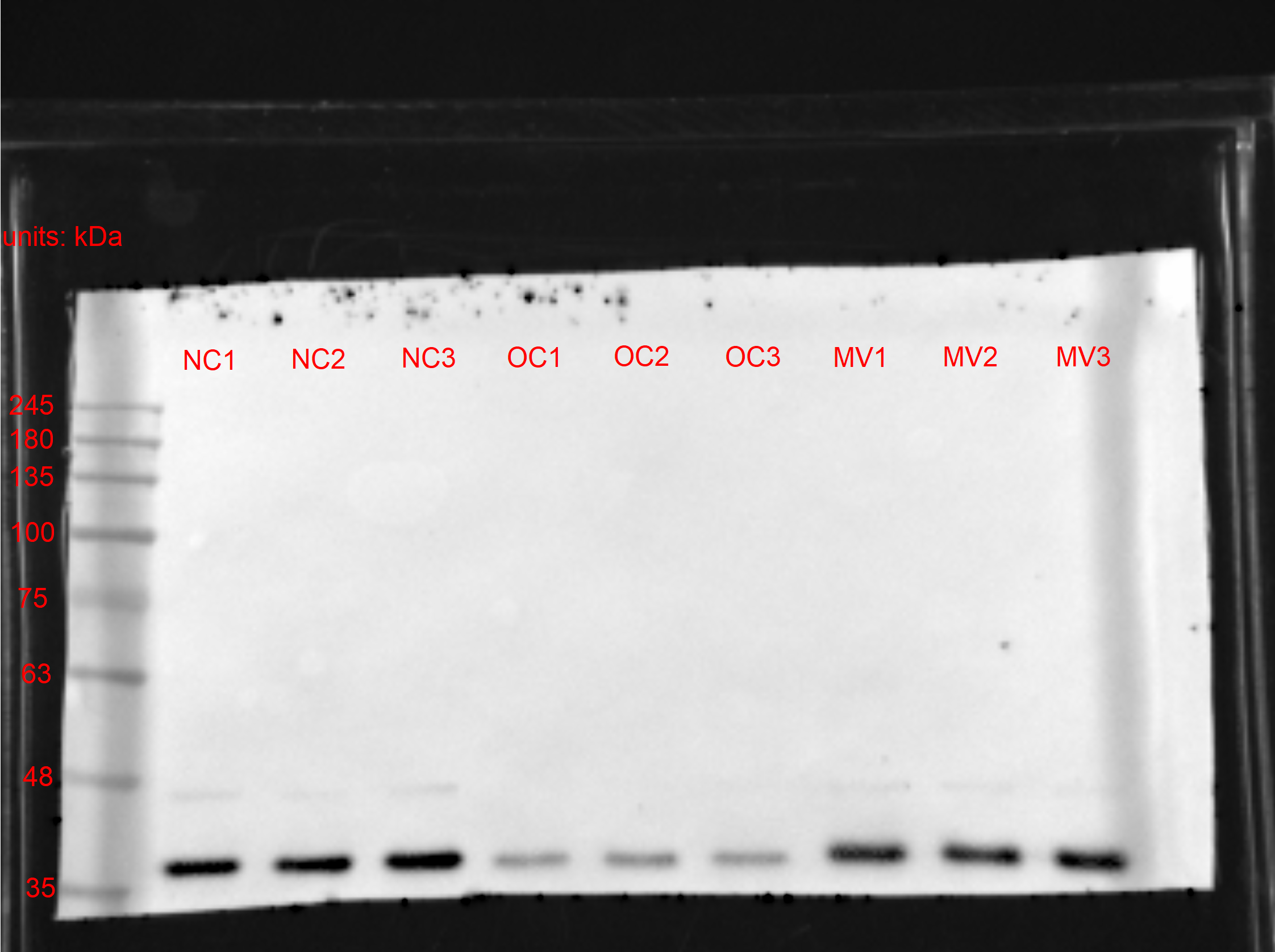

Supplement: Supplementary file 1 [file DataSheet_1.zip › WB╘¡╩╝╩2╛▌-TIFF/IkB----HB-.tif]

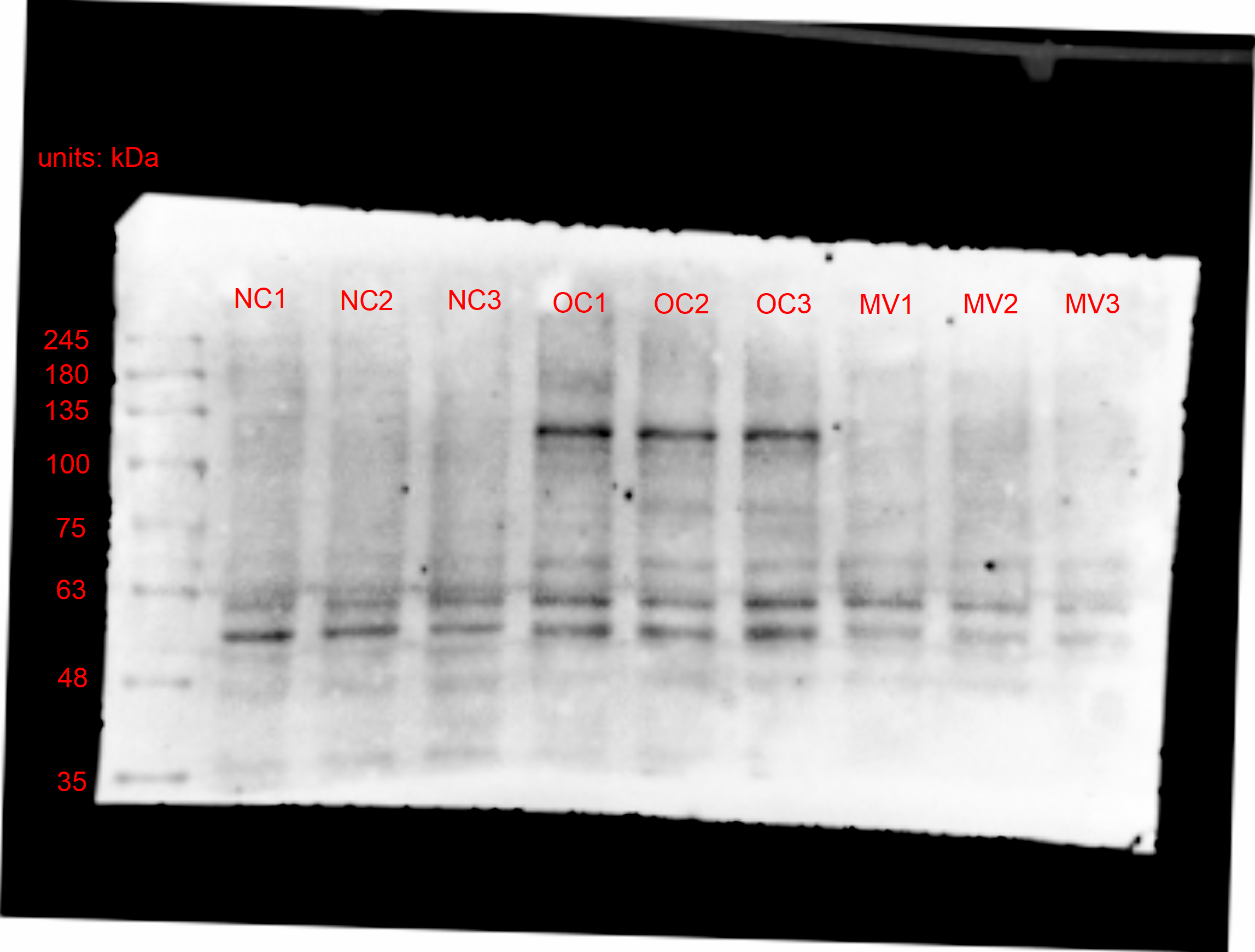

Supplement: Supplementary file 1 [file DataSheet_1.zip › WB╘¡╩╝╩2╛▌-TIFF/JAK-----HB-.tif]

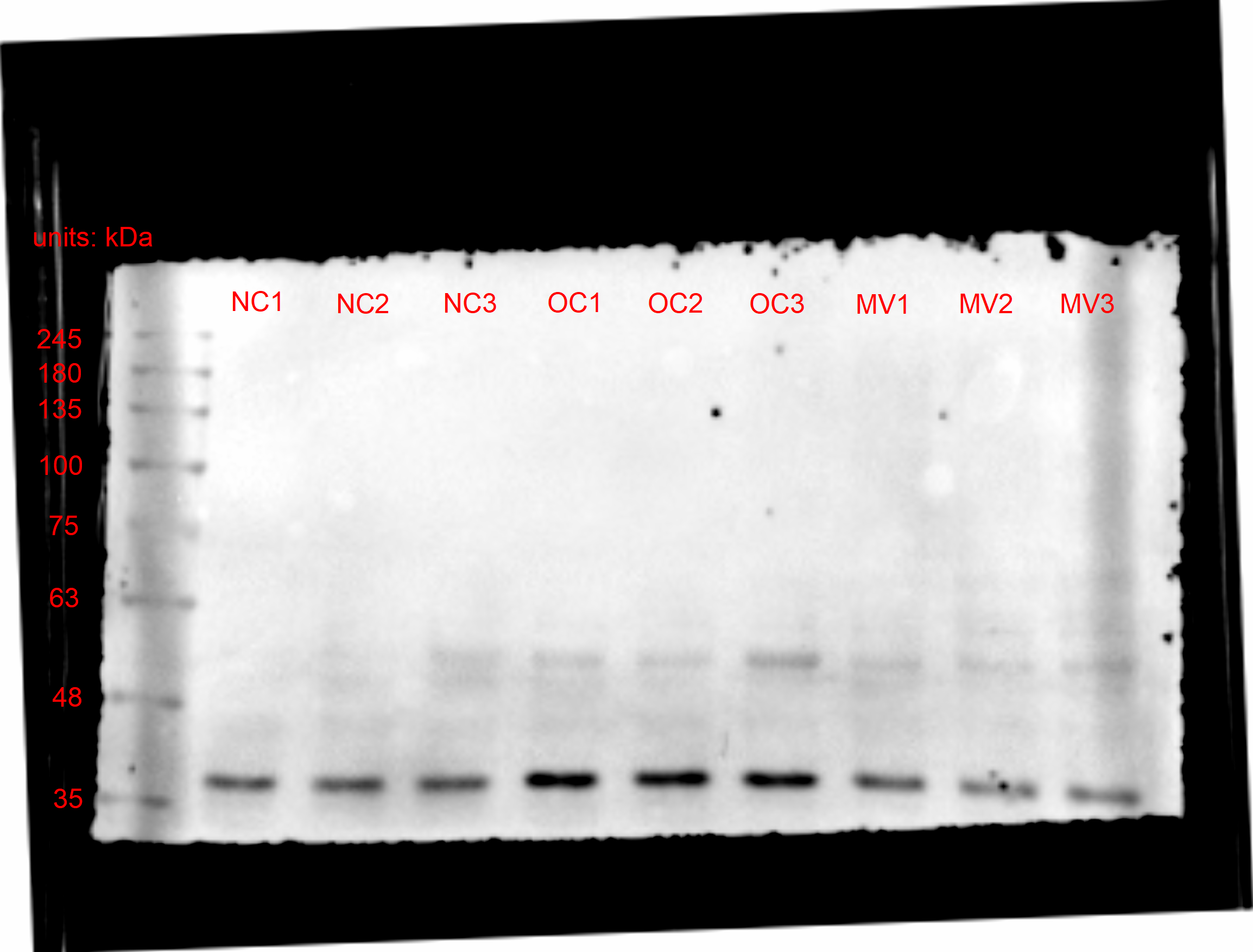

Supplement: Supplementary file 1 [file DataSheet_1.zip › WB╘¡╩╝╩2╛▌-TIFF/P-IkB---HB-.tif]

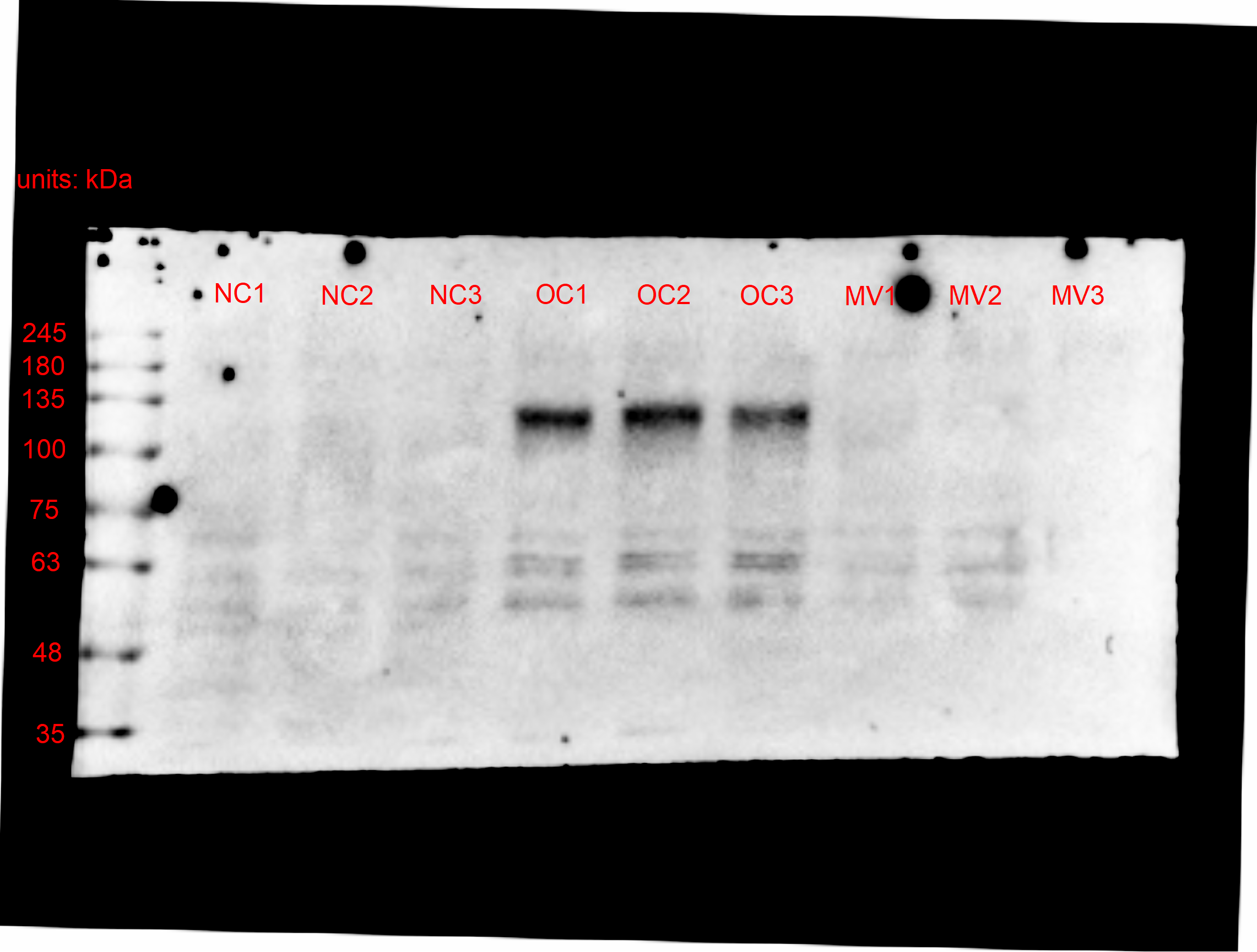

Supplement: Supplementary file 1 [file DataSheet_1.zip › WB╘¡╩╝╩2╛▌-TIFF/P-JAK----HB-.tif]

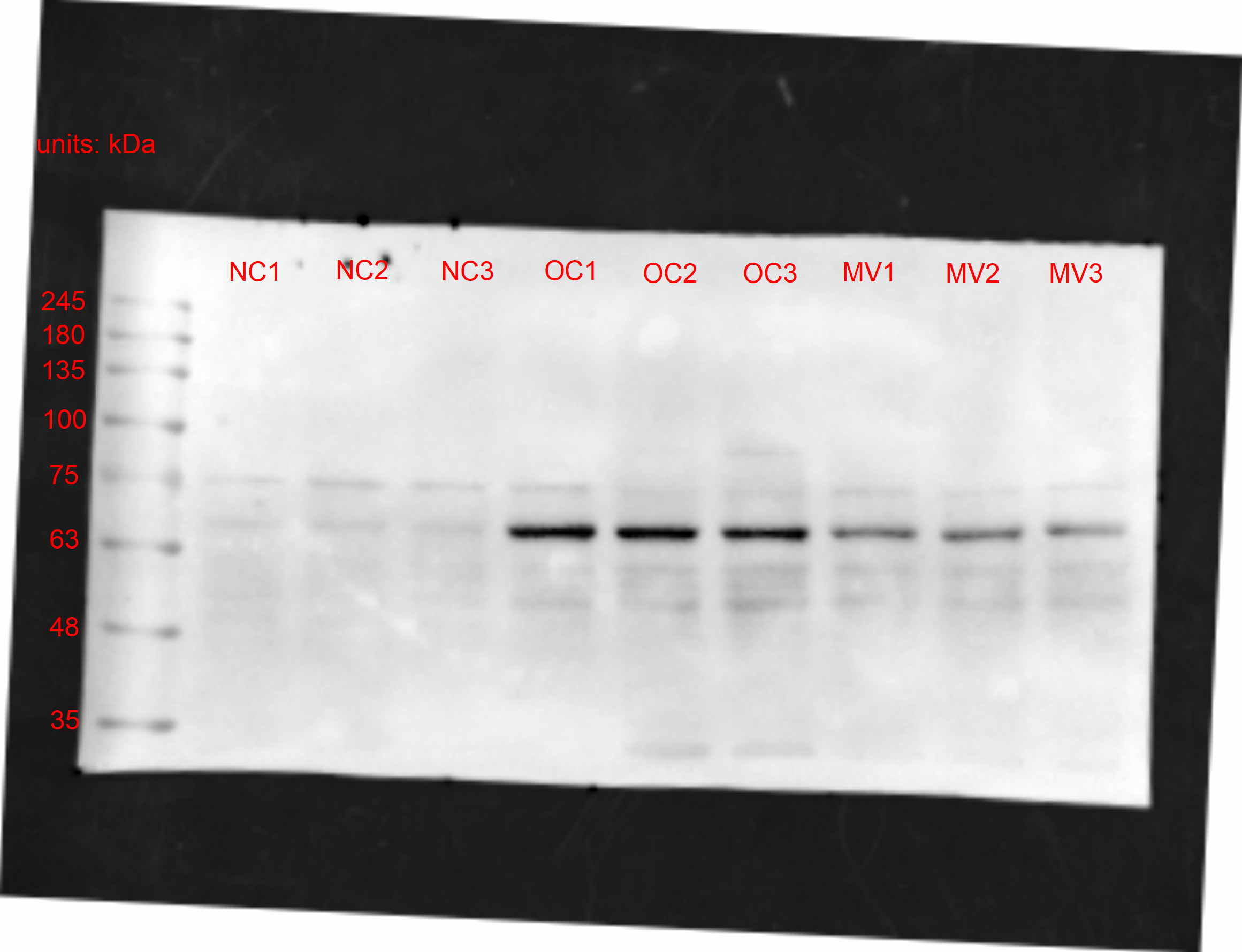

Supplement: Supplementary file 1 [file DataSheet_1.zip › WB╘¡╩╝╩2╛▌-TIFF/P-P65----HB-.tif]

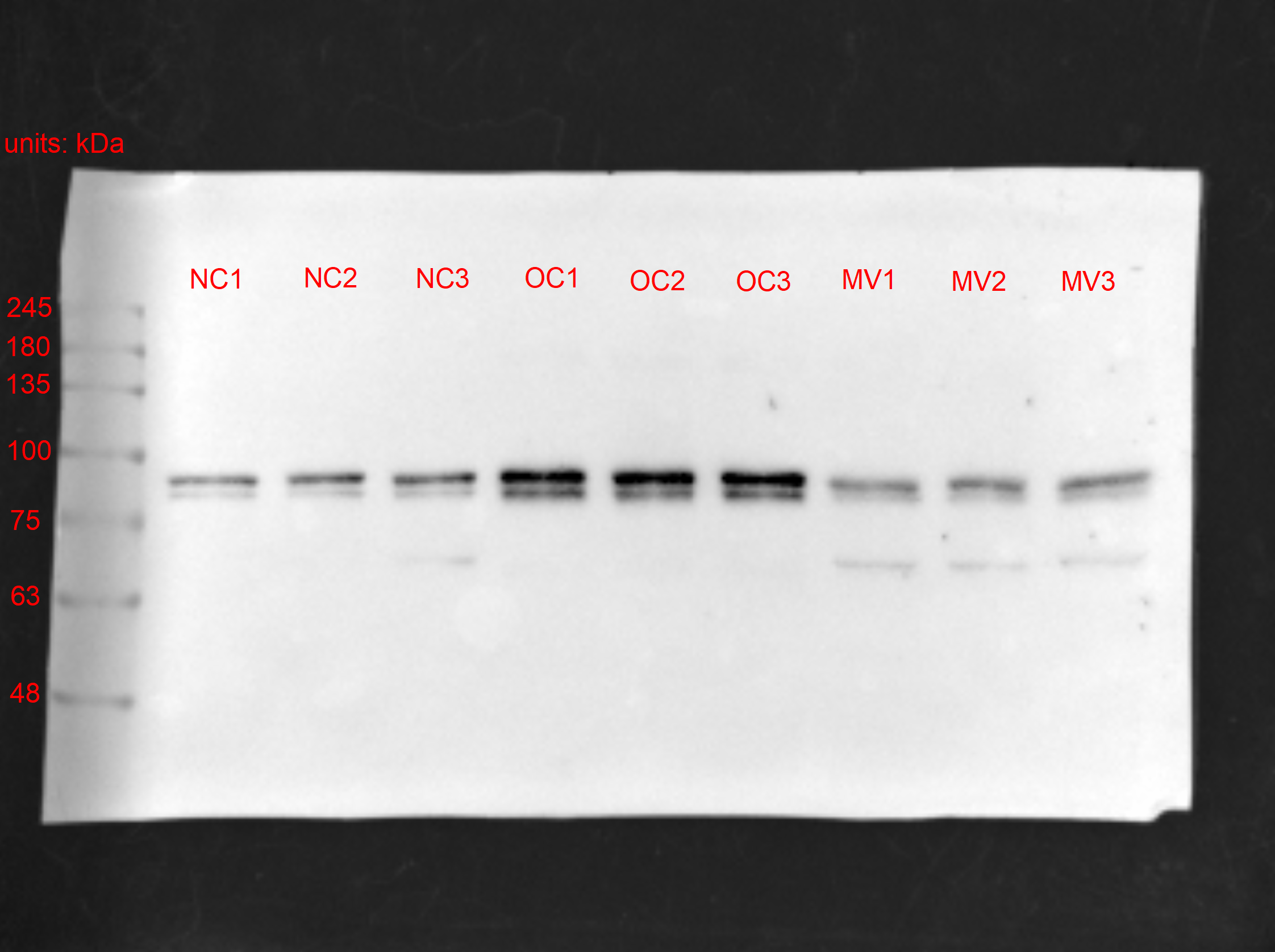

Supplement: Supplementary file 1 [file DataSheet_1.zip › WB╘¡╩╝╩2╛▌-TIFF/P-Stat1---HB-.tif]

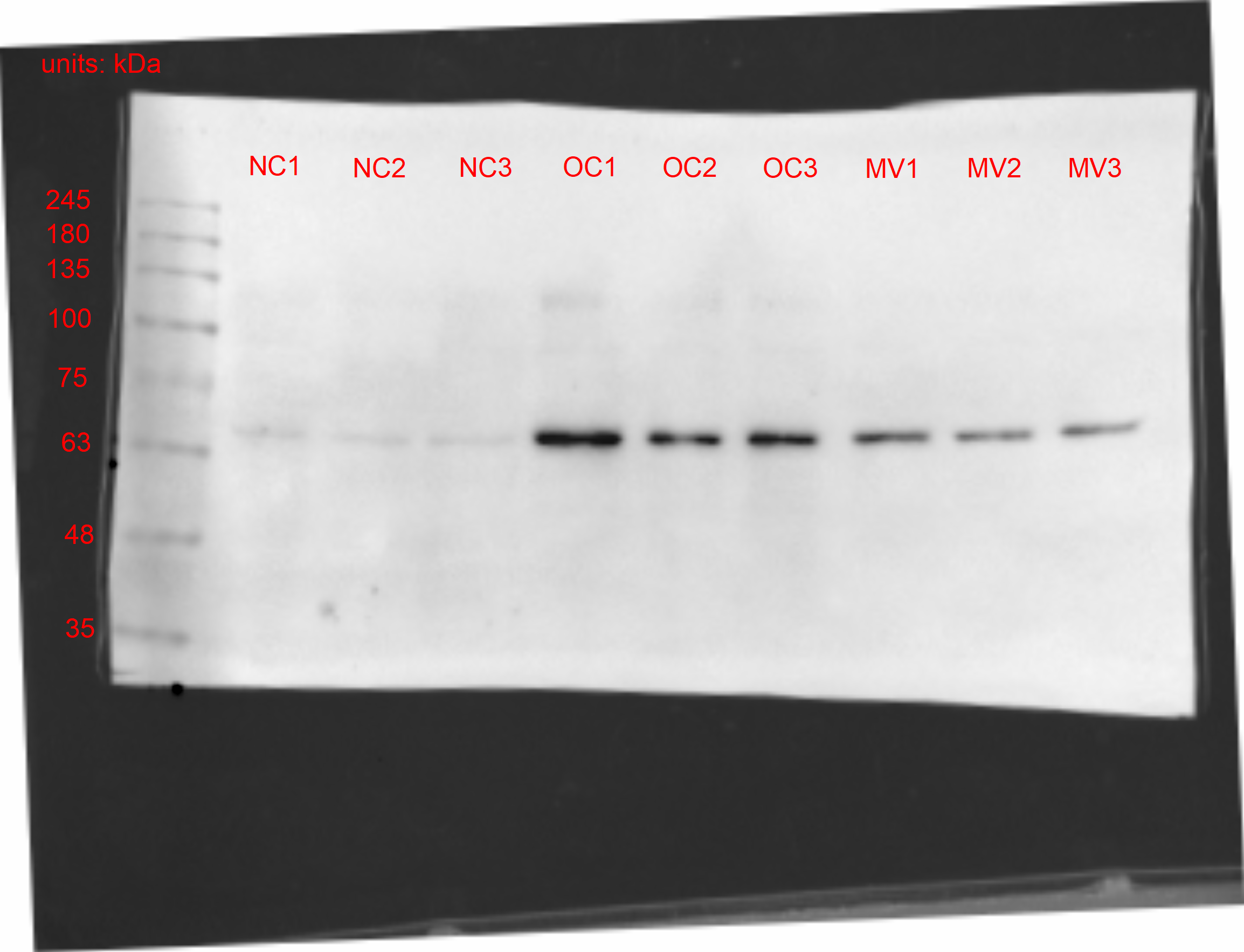

Supplement: Supplementary file 1 [file DataSheet_1.zip › WB╘¡╩╝╩2╛▌-TIFF/P65-----HB-.tif]

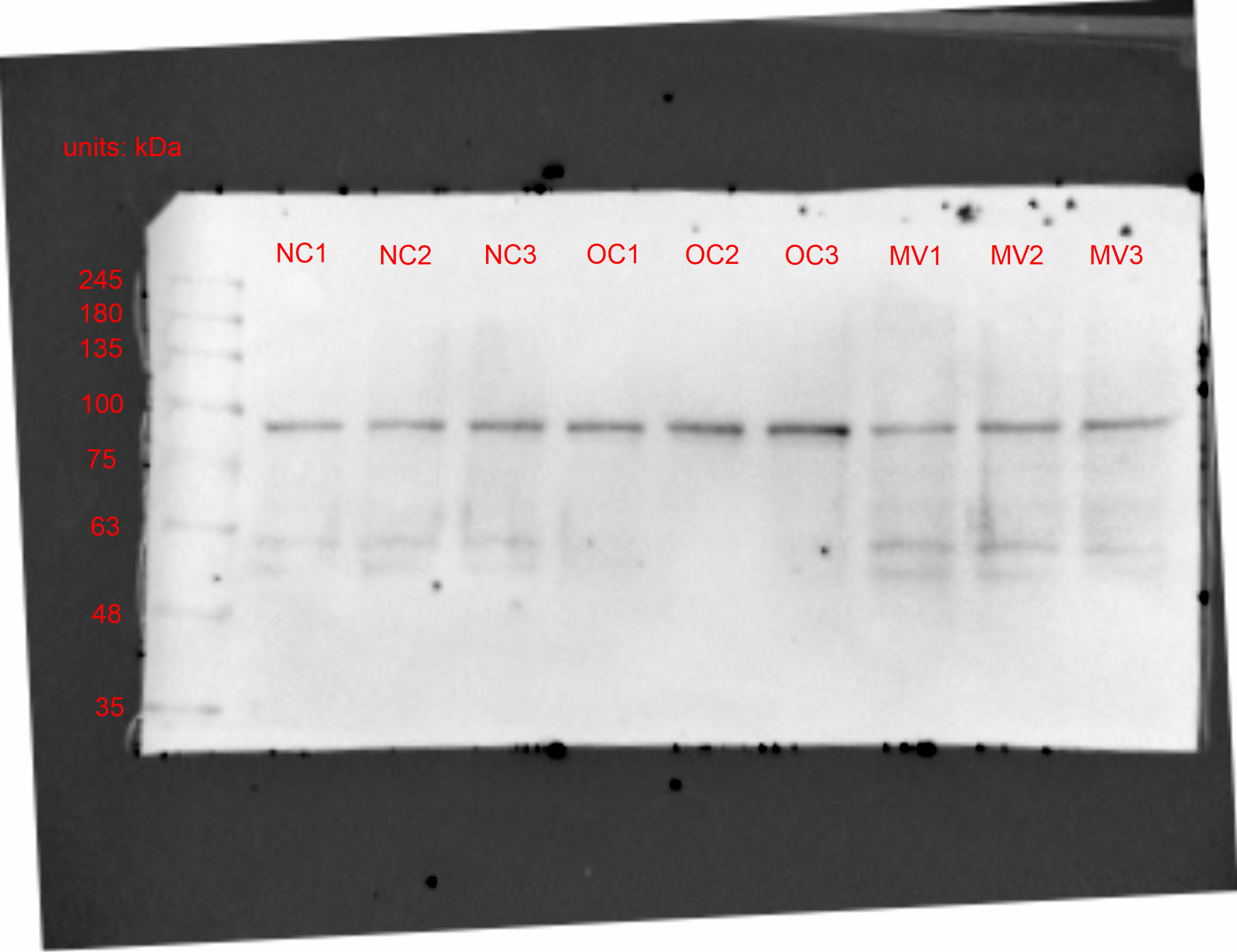

Supplement: Supplementary file 1 [file DataSheet_1.zip › WB╘¡╩╝╩2╛▌-TIFF/Stat1-----HB-.tif]

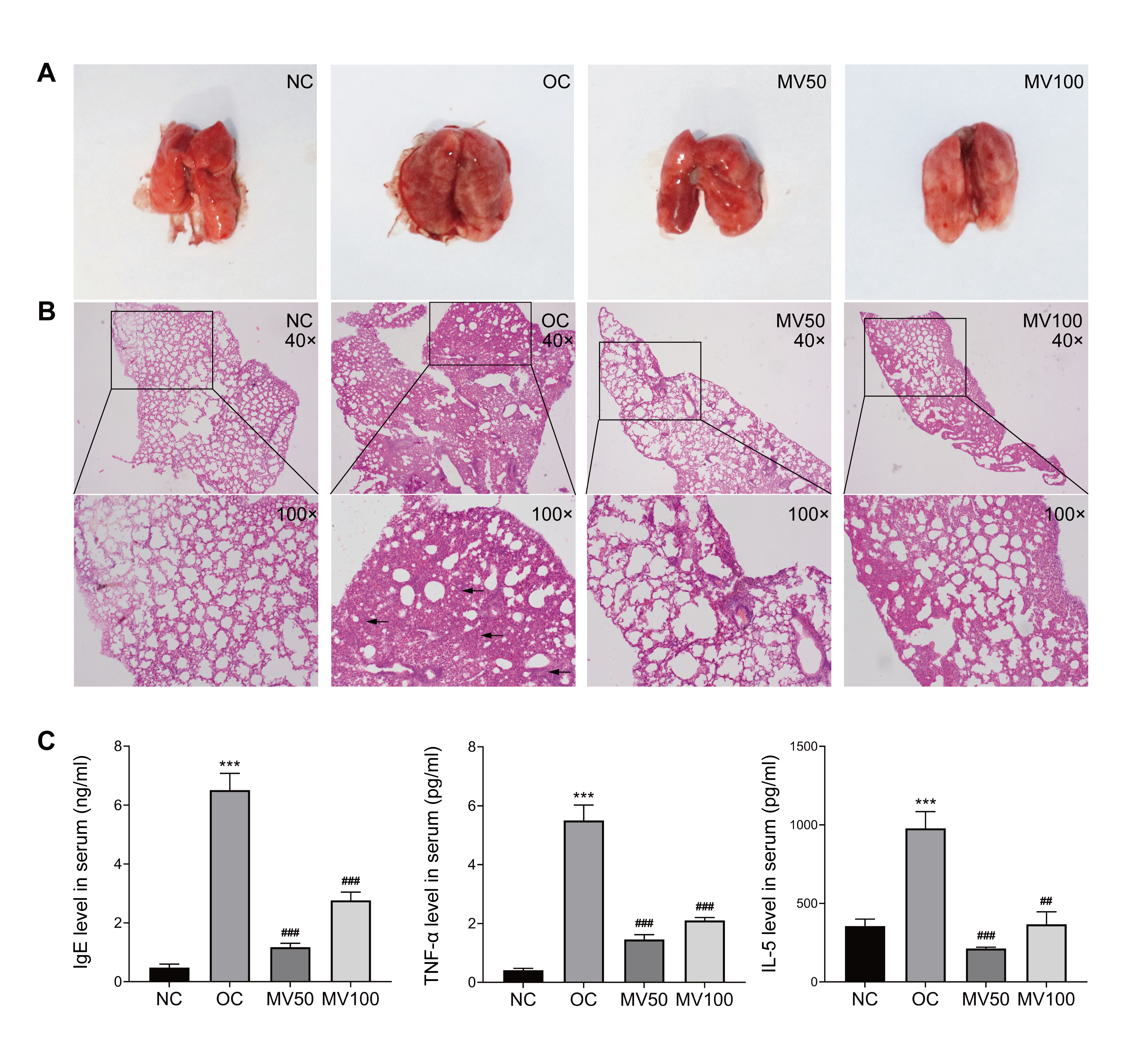

Supplement: Supplementary file 4 [file Image_1.jpeg]
